# Music-based emotion regulation: a bibliometric systematic review (2000–2024)

**DOI:** 10.3389/fpsyg.2025.1565614

**Published:** 2025-10-14

**Authors:** Xixi Lu, Shijie Song

**Affiliations:** ^1^School of Music, Chengdu Normal University, Chengdu, China; ^2^Department of Sports Science, Beijing Sport University, Beijing, China

**Keywords:** music emotion regulation, bibliometric analysis, research trends, knowledge structure, cross-disciplinary integration, scientific collaboration, mental health intervention

## Abstract

**Background:**

Music Emotion Regulation (MER) research has emerged as a significant field in mental health intervention, yet a comprehensive bibliometric analysis of its development and current status is lacking.

**Purpose:**

This research aims to systematically analyze the knowledge structure, research trends, and development patterns in MER research through bibliometric analysis.

**Methods:**

Using data from the Web of Science Core Collection (2000–2024), we conducted a comprehensive bibliometric analysis using R for primary analyses. We examined publication trends, collaboration networks, research themes, and emerging directions through multiple bibliometric indicators including co-citation analysis, keyword co-occurrence, and research theme evolution.

**Results:**

The analysis revealed three distinct development phases of MER research: initial exploration (2000–2009), rapid growth (2010–2019), and maturation (2020–2024). Research in the field demonstrates a clear transition from theoretical foundations to practical applications, with increasing methodological sophistication and cross-disciplinary integration. International collaboration networks show a “tri-polar” pattern centered in East Asia, Europe, and North America, with distinctive regional research characteristics.

**Conclusion:**

MER research has evolved into a mature, interdisciplinary field with robust theoretical foundations and practical applications. Future development trends point toward increased integration of artificial intelligence, personalized intervention strategies, and expanded international collaboration.

## 1 Introduction

Mental health issues have emerged as a major public health challenge in contemporary society, with their severity and scope of impact continuously expanding. Epidemiological investigations indicate that the lifetime prevalence of mental disorders globally exceeds 20% (Nochaiwong et al., 2021), with a significant upward trend in the incidence of common psychological disorders (Wu et al., 2023) such as depression and anxiety. Among various mental health intervention strategies, music, as a non-pharmacological therapeutic approach, has become a research focus in the mental health field due to its distinctive advantages including non-invasiveness, high compliance, and cost-effectiveness. Empirical evidence demonstrates that music intervention, through its unique emotion regulation mechanisms, can effectively modulate negative emotional experiences and facilitate positive emotional responses, thereby enhancing psychological well-being (Gustavson et al., 2021). In clinical psychological practice, music therapy, as a systematic adjunctive therapeutic approach, has demonstrated significant applicational value in the prevention and intervention of emotional disorders (Hegde, 2017), providing new research paradigms and practical approaches for mental health promotion.

It is essential to distinguish Music Emotion Regulation (MER) from music therapy to clarify the scope of this bibliometric analysis. MER refers to the intentional use of music by individuals themselves as a self-directed strategy to influence, modify, or manage their emotional states. This encompasses both conscious and unconscious processes whereby people select, engage with, or create music to achieve desired emotional outcomes, such as mood enhancement, stress reduction, or emotional expression. In contrast, music therapy involves structured therapeutic interventions delivered by trained and certified music therapists within clinical or educational settings, following established therapeutic protocols and goals. While music therapy represents a professional healthcare service with specific training requirements and clinical frameworks, MER encompasses the broader phenomenon of everyday music use for emotional purposes by the general population. This distinction is crucial as our analysis focuses on research examining music’s role in emotion regulation across various contexts, including but not limited to clinical applications, rather than being restricted to formal therapeutic interventions alone.

In recent years, research on Music Emotion Regulation (MER) has demonstrated substantial growth, with continuous advancement in research perspectives and methodologies. Researchers have conducted extensive investigations into the mechanisms of MER, regulatory effectiveness evaluation, and its applications across various emotional states. Existing studies indicate that MER demonstrates significant efficacy in ameliorating negative emotions (Leubner and Hinterberger, 2017), enhancing emotion regulation capabilities (Chong et al., 2024), and promoting psychological well-being (Viola et al., 2023). Although several systematic reviews and meta-analyses have focused on MER research (Peters et al., 2024), these studies are primarily limited to clinical perspectives and predominantly emphasize randomized controlled trials in their literature selection. With the diversification of MER research, focusing solely on a single research paradigm is insufficient to comprehensively grasp the developmental trajectory of MER research. In contrast, bibliometric analysis can reveal the interconnections between different research paradigms, effectively handle heterogeneous studies, and identify potential research gaps and emerging directions based on large-scale literature data (Ninkov et al., 2022). However, there remains a lack of systematic analysis from a bibliometric perspective regarding the evolution of research hotspots, knowledge foundation composition, and future development directions in this field. Furthermore, while a recent scoping review by Chong et al. (2024) provided valuable insights into music emotion regulation research, their study was limited by challenges in comprehensive literature identification and conceptual boundary definition. A bibliometric analysis can complement and extend their findings by providing a more systematic and quantitative perspective on the field’s development.

Based on the aforementioned research landscape, the knowledge map of MER research was systematically examined using bibliometric analysis. Specifically, this research aims to reveal the evolutionary patterns of research hotspots, the distribution and interconnections of core themes, characteristics of international collaboration networks, and emerging research trends in this field. Through systematic analysis of relevant literature from the Web of Science core database, present study will delineate the overall knowledge structure of MER research, thoroughly explore its application prospects in mental health promotion, and provide reference for future theoretical construction and practical development in this field. This research not only helps researchers grasp the development dynamics of the field but also provides new insights and directions for mental health intervention practices.

## 2 Materials and methods

### 2.1 Research tools

Primary analyses were conducted using R 4.3.3, with specialized bibliometric packages for literature analysis and visualization. The analytical workflow, including data processing scripts and raw bibliometric data, was made available in the Supplementary Appendix for reproducibility. For validation and complementary analysis, CiteSpace 6.4.R1 was also employed to generate supplementary visualizations of citation networks and research themes, with these results included in the Supplementary Appendix.

### 2.2 Conceptualization and operational definition of MER

This study employed bibliometric methods to investigate the knowledge mapping of MER, which necessitated a clear delineation of its conceptual boundaries and operational definitions. In terms of conceptual clarification, MER is distinct from general Music Emotion (ME) research, as it specifically emphasizes the functional application of music as an emotion regulation strategy. This distinction is crucial for ensuring the precision of retrieval strategies and the clarity of the scope of the study.

**FIGURE 1:**
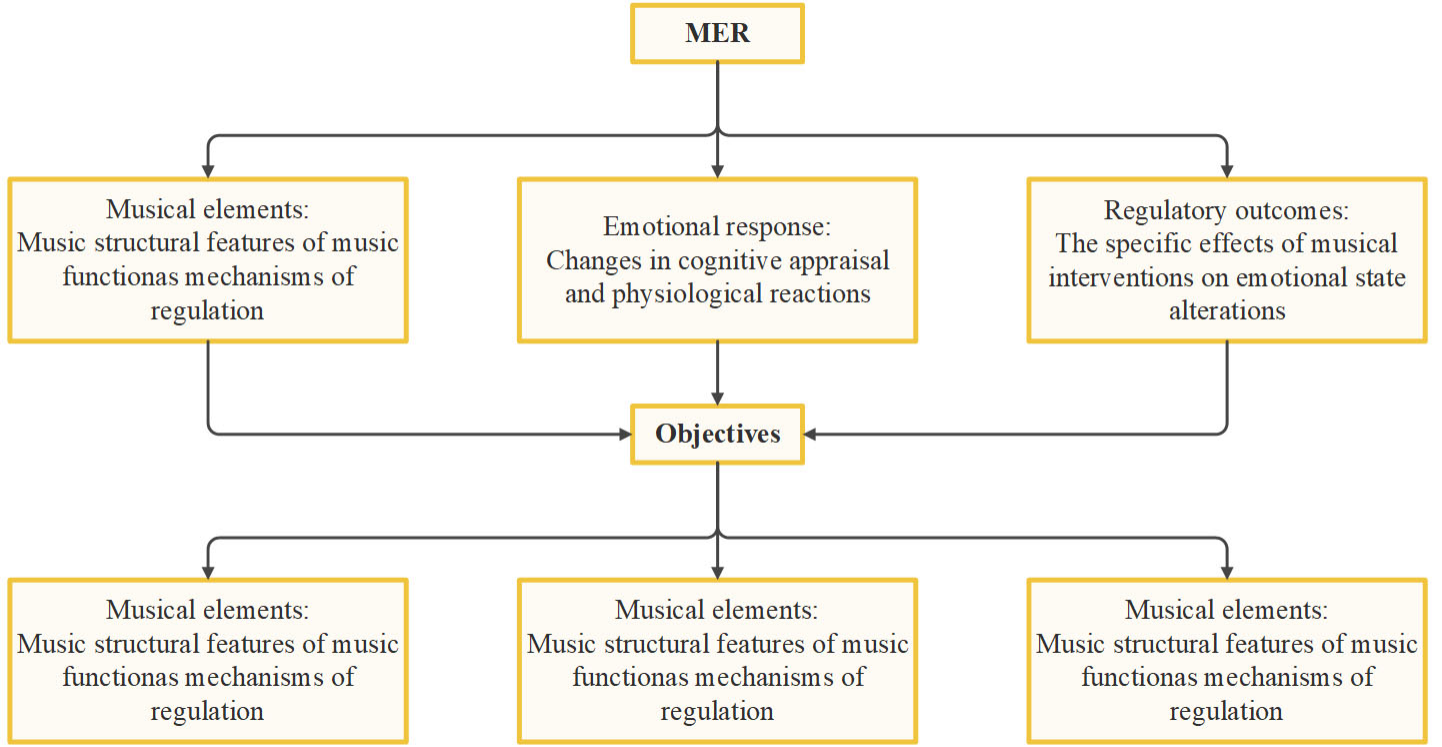
MER framework.

Based on the objectives of the current study and following the theoretical framework proposed by Chong et al. (2024), as shown in Figure 1, MER is operationalized across three dimensions: First, at the level of musical elements, the focus is on how structural features of music (e.g., rhythm, melody, harmony) function as mechanisms of regulation. Second, at the level of emotional responses, attention is directed to changes in cognitive appraisal and physiological reactions. Lastly, at the level of regulatory outcomes, the emphasis is placed on examining the specific effects of musical interventions on emotional state alterations. This framework guided our literature search and analysis.

To systematically grasp the landscape of MER research, this study adopts Boolean operations to construct a retrieval strategy in the selected databases, ensuring the acquisition of comprehensive and relevant literature samples. We designed the search strategy based on this three-dimensional framework, using keywords related to musical intervention, emotional response, and regulatory outcomes. This conceptually framed retrieval strategy design enhances the accuracy and completeness of the literature collection process.

### 2.3 Data sources

**FIGURE 2:**
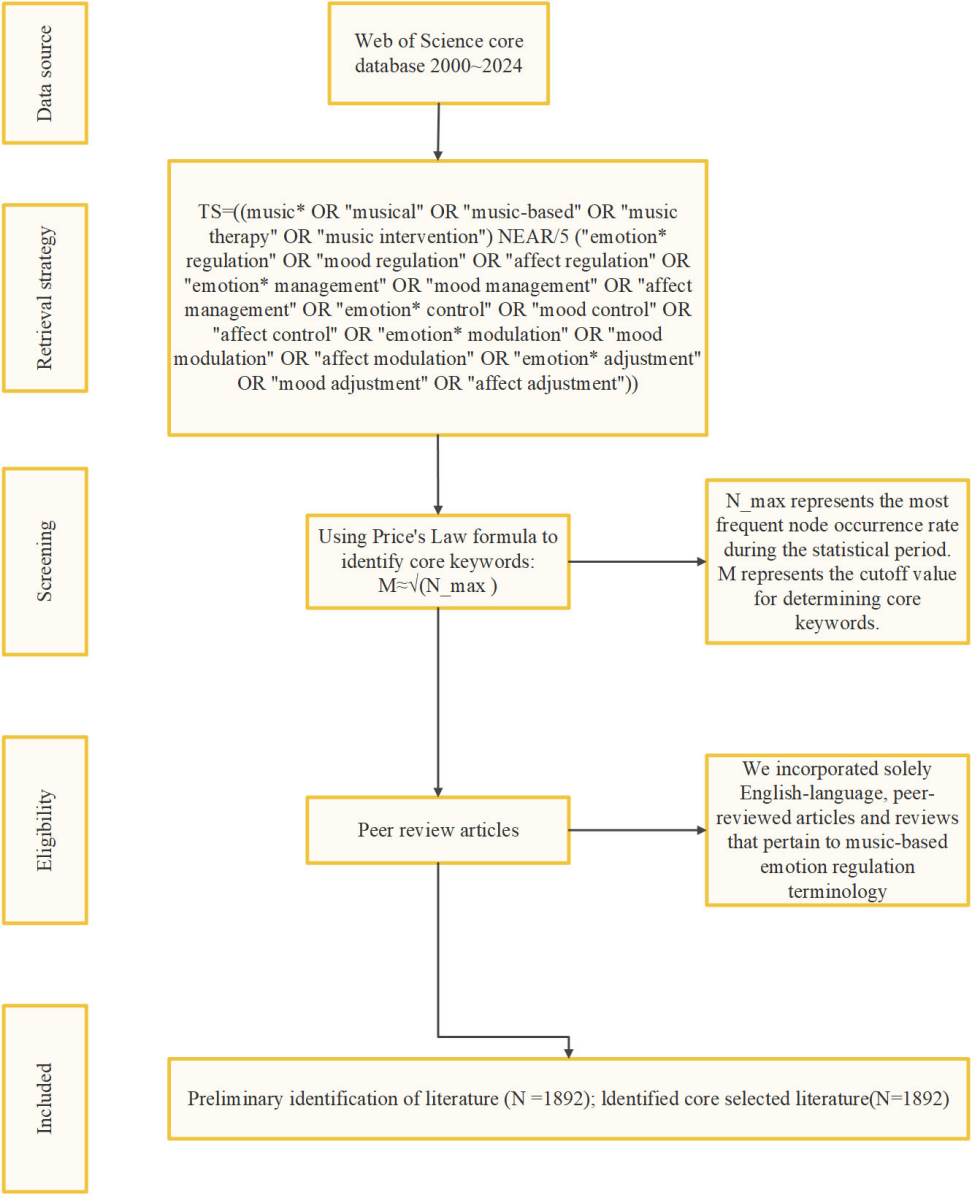
Retrieval strategy.

Data were extracted from the Web of Science Core Collection (Science Citation Index Expanded and Social Sciences Citation Index), spanning from 2000 to 2024. The search strategy (Refer to Supplementary Appendix A) incorporated terms related to music-based emotion regulation, yielding a comprehensive dataset of relevant publications. To ensure analytical rigor and accessibility, only peer-reviewed articles and reviews published in English were included in the analysis. The retrieval strategy as shown in Figure 2.

### 2.4 Identification of core keywords

We identified core keywords using Price’s Law (Price, 2019), expressed as Equation 1:


(1) $$M\approx \sqrt{N_{m\,a\,x}}$$

Here, *N*_max_ represents the frequency of the most commonly occurring node during the statistical period, and *M* denotes the threshold value for core keywords. A node is considered a core node if its occurrence frequency *f* satisfies the condition, which has the mathematical expression as shown in Equation 2:


(2) f≥M

This adaptation of Price’s Law enabled the identification of pivotal terms within the field, highlighting the keywords with significant influence. The approach ensured that core nodes are selected based on their contribution frequency in relation to the statistical maximum. Notably, all statistical results in this investigation are presented without rounding, preserving accuracy.

## 3 Results and analysis

### 3.1 Bibliometric overview

#### 3.1.1 Publication trends and impact analysis

**FIGURE 3:**
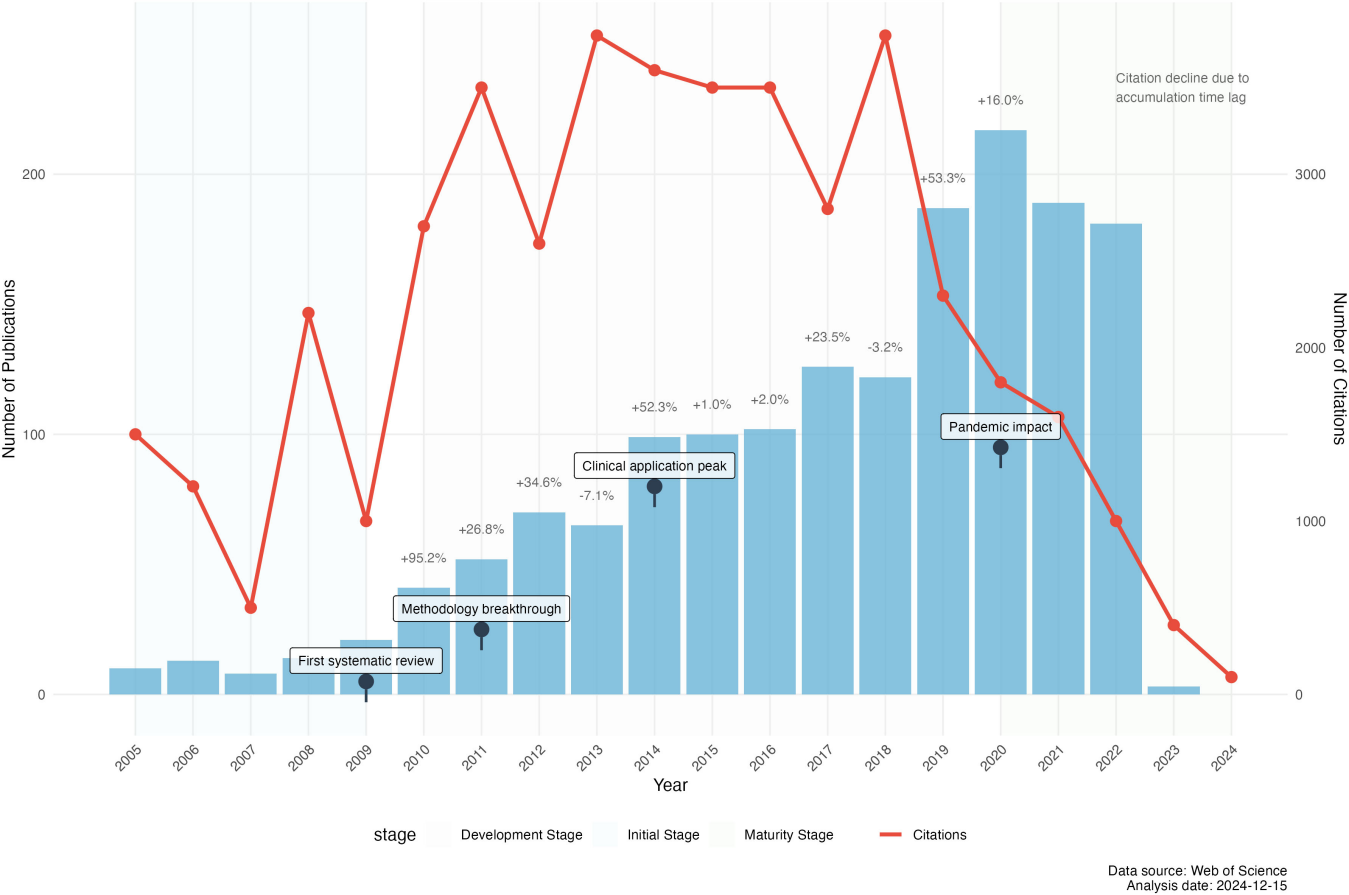
Annual publications and citations in MER research (2000–2024).

The bibliometric analysis identified 1,819 MER publications from 2000 to 2024, accumulating 40,814 citations (average citations per paper: 22.44). The field’s development demonstrated three distinct phases: initial exploration (2000–2009), rapid growth (2010–2019), and maturation (2020–2024). The annual publication volume increased from 13 papers in 2000 to over 180 papers during 2021–2023, with a compound annual growth rate of 16.8%. The annual publication and citation demonstration as shown in Figure 3. Papers from 2008 to 2013 had the highest citation rates (35.7–154 per paper), with 2008 papers averaging 154 citations, indicating foundational contributions. The top 10 highly cited papers in MER research are listed in Table 1, with complete author information, journal details, and research theme classification criteria provided in Supplementary Tables 1, 2 and Supplementary Information (see Supplementary Appendix).

**TABLE 1:** Top 10 highly cited papers in MER research (sorted by citations in descending order).

Rank	First author	Year	Citations	Journal	Research theme
**Theoretical foundations (TF)**					
1	Juslin PN*	2008	1003	Behavioral and brain sciences	Theoretical foundations
**Neural mechanisms (NM)**					
2	Menon V*	2005	634	NeuroImage	Neural mechanisms
3	Hong KS*	2015	367	Frontiers in human neuroscience	Neural mechanisms
4	Zentner M*	2010	304	PNAS	Neural mechanisms
**Methodological innovation (MI)**					
5	Chanda ML*	2013	505	Trends in cognitive sciences	Methodological innovation
**Clinical applications (CA)**					
6	Särkämö T*	2008	499	Brain	Clinical applications
7	Carlson LG*	2017	493	CA: A cancer journal for clinicians	Clinical applications
**Emotional processing (EP)**					
8	Koelsch S*	2010	388	Trends in cognitive sciences	Emotional processing
9	Robins KJ*	2010	326	Emotion	Emotional processing
10	Lehrner J*	2005	272	Physiology and behavior	Emotional processing

Complete author lists available in Supplementary material; Correspondence authors marked with asterisk.

#### 3.1.2 Geographic distribution and regional characteristics

**FIGURE 4:**
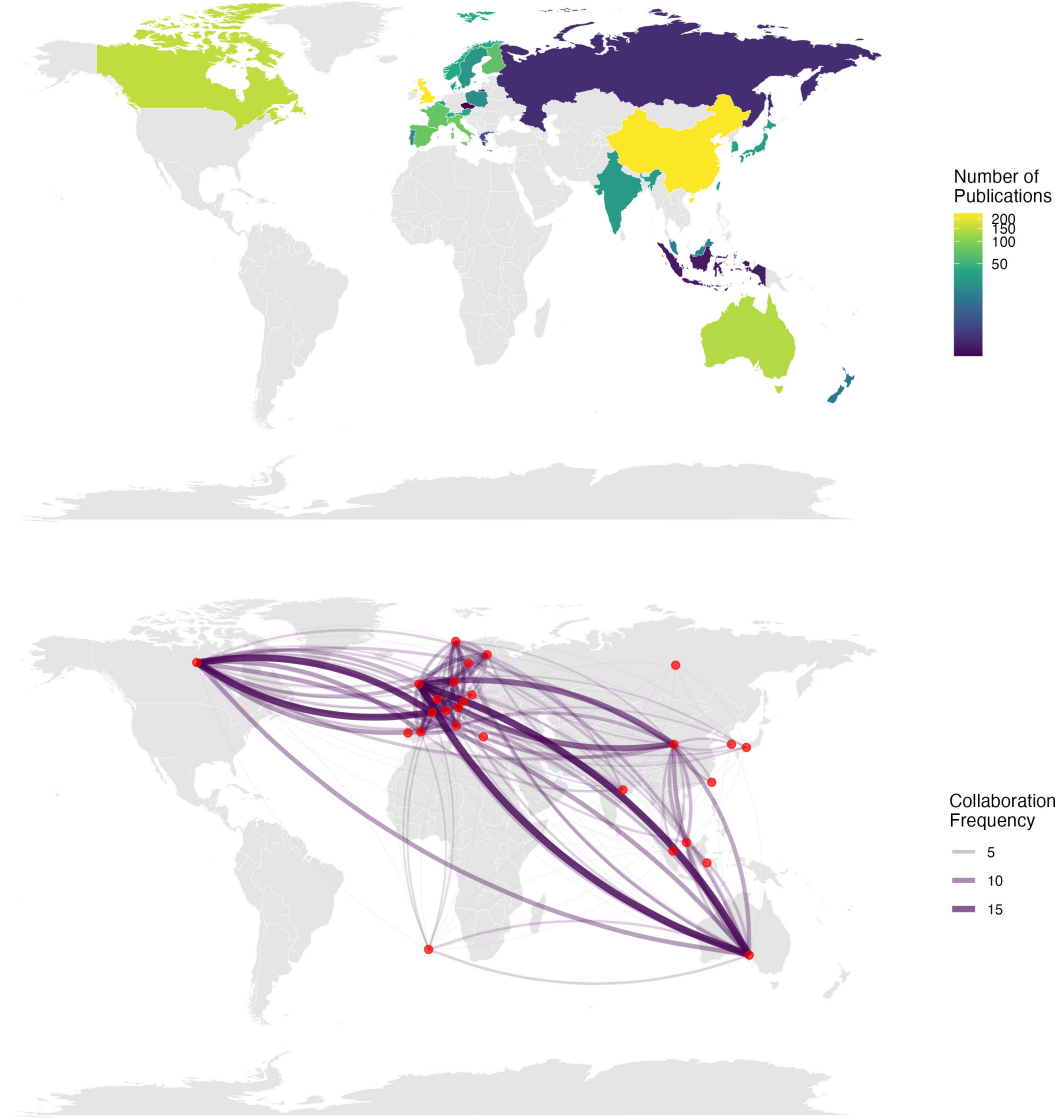
Global distribution and collaboration in MER research.

The geographic distribution analysis reveals a “tri-polar” pattern centered in East Asia, Europe, and North America. As shown in Table 2, which presents the regional research performance metrics, China leads in publication volume (234 articles, 12.86%), followed by the United Kingdom (218 articles, 11.98%) and Germany (171 articles, 9.40%). Impact analysis shows distinct regional characteristics - while Western European countries demonstrate higher citation impact (e.g., UK: 6,533 citations; Germany: 4,563 citations), East Asian contributions focus on volume output. Nordic countries, despite lower publication counts, show remarkable citation efficiency (Finland: 48.21 citations/paper; Sweden: 71.67 citations/paper). The map of global distribution and collaboration in MER research as shown in Figure 4.

**TABLE 2:** Regional research performance metrics (sorted by total publications in descending order).

Country	Total publications	Citations	Citations per Paper	H-index	Collaboration ratio	NCII
UK	218	6,533	29.97	35	50.20%	1.344
China	234	1,769	7.56	36	23.50%	0.339
Germany	171	4,563	26.69	35	49.70%	1.198
Canada	152	3,339	21.97	29	47.40%	0.854
Australia	139	3,023	21.75	29	41.70%	0.845
Italy	84	2,344	27.9	25	45.20%	1.084
Spain	78	3,001	38.47	26	35.90%	1.495
France	74	1,586	21.43	22	60.80%	0.833
Finland	69	2,283	33.09	24	56.50%	1.286
Netherlands	64	1,589	24.83	22	60.90%	0.965
Austria	42	1,298	30.9	13	66.70%	1.201
Switzerland	42	1,397	33.26	16	71.40%	1.293
Norway	41	1,867	45.54	19	75.60%	1.77
South Korea	38	606	15.95	12	26.30%	0.62
Taiwan	37	731	19.76	14	37.80%	0.768
Belgium	36	928	25.78	13	77.80%	1.002
Denmark	36	747	20.75	13	75.00%	0.807
Brazil	35	936	26.74	16	34.30%	1.039
Israel	35	861	24.6	16	40.00%	0.956
Japan	33	491	14.88	12	36.40%	0.578

#### 3.1.3 Journal distribution and disciplinary coverage

**FIGURE 5:**
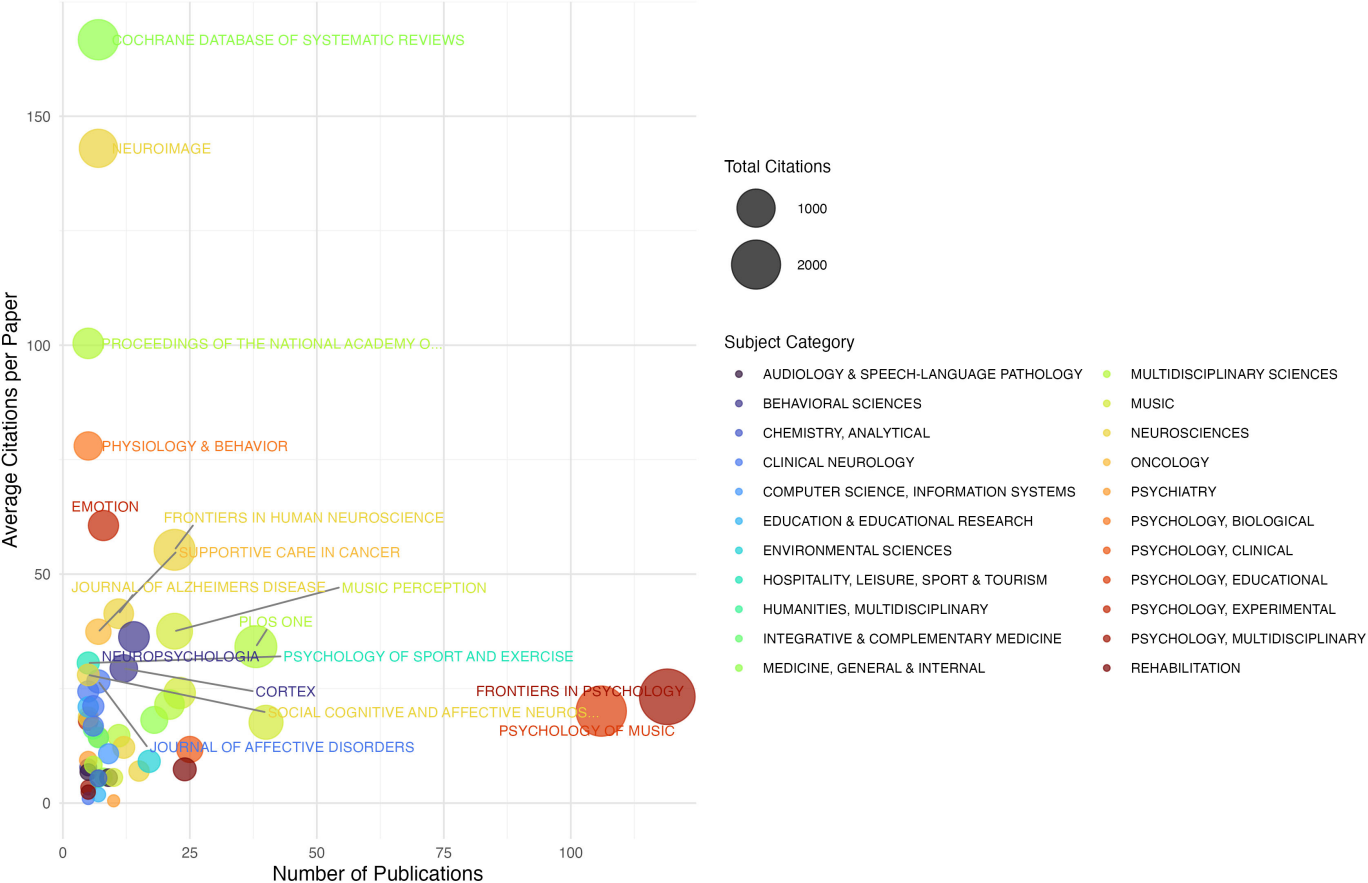
Journal impact and disciplinary coverage in MER research.

**FIGURE 6:**
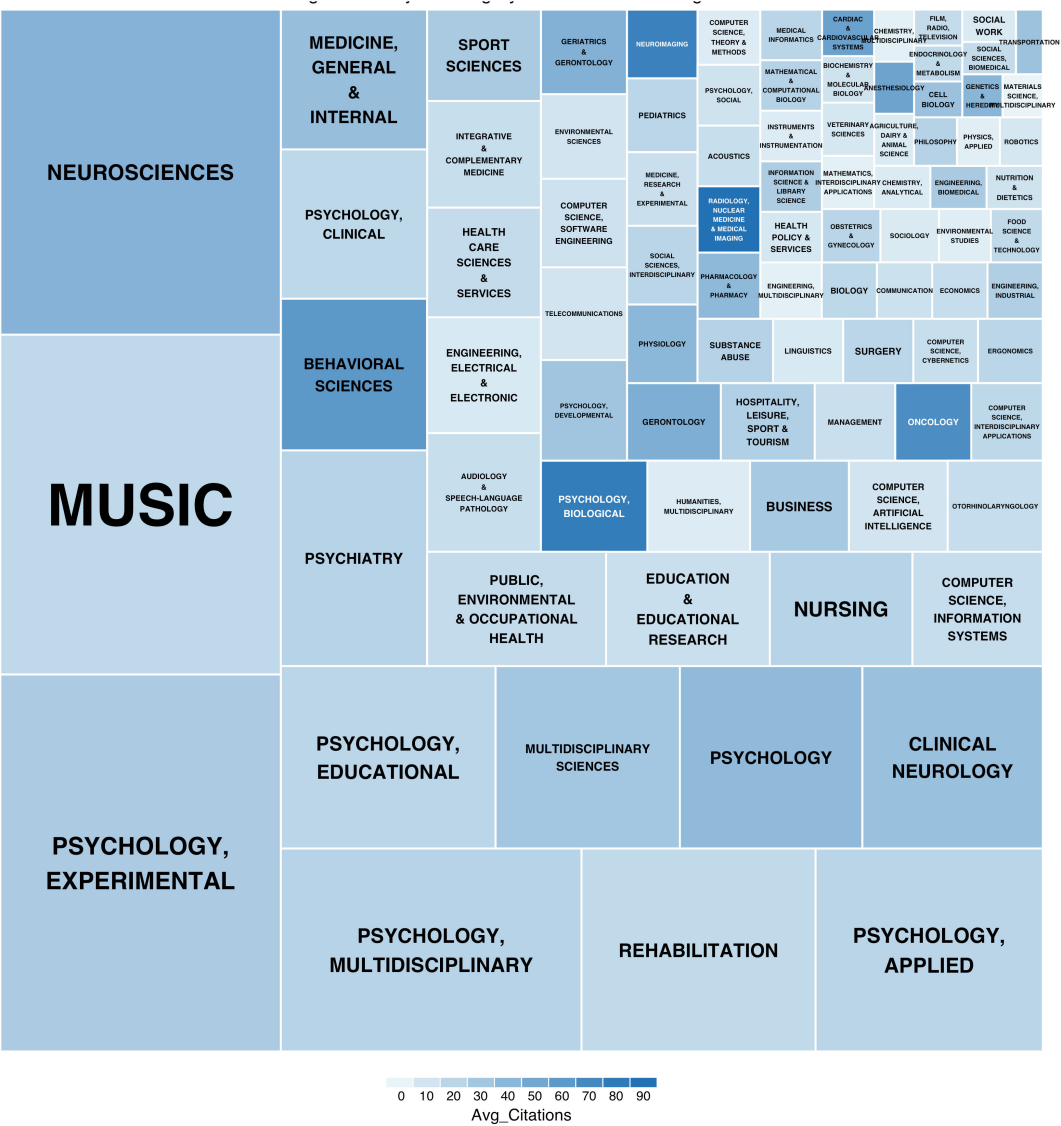
Subject category distribution and integration.

Publication outlets demonstrate clear stratification, with 15 core journals accounting for 60.3% of total publications. The field is primarily anchored in psychology and neuroscience journals, with Frontiers in Psychology (119 articles) and Psychology of Music (106 articles) leading in volume. Impact analysis identifies differential patterns - specialized journals like Frontiers in Human Neuroscience show higher citation impact (55.32 citations/article), while general psychology journals contribute to broader dissemination. The disciplinary analysis reveals substantial interdisciplinarity, with 44.04% of papers involving multiple disciplines. The core disciplinary triangle comprises experimental psychology (305 articles), musicology (275 articles), and neuroscience (263 articles), forming distinct but interconnected research communities (modularity = 0.571). The demonstration of journal impact and disciplinary coverage in MER research as shown in Figure 5. The illustration of subject category distribution and integration as shown in Figure 6.

#### 3.1.4 Research methodology profile

**FIGURE 7:**
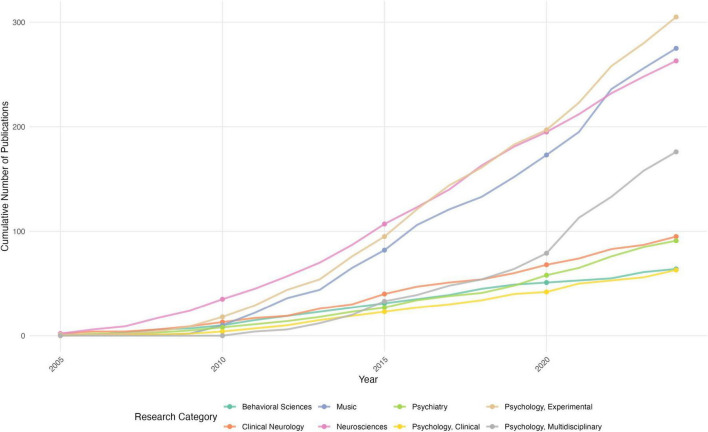
Evolution of research methods in MER studies.

Methodological analysis demonstrates the field’s empirical orientation, with experimental studies dominating (52.3%, *n* = 951), followed by observational studies (31.7%, *n* = 577) and theoretical works (16.0%, *n* = 291). The distributions and applications of research methodology are shown in Table 3. Temporal analysis shows methodological evolution - while early studies (2000–2009) relied heavily on behavioral measures, recent research (2020–2024) increasingly incorporates physiological measurements (23.7%) and mixed methods (18.4%). Measurement approaches show clear stratification: subjective assessments lead (65.2%), followed by physiological measures (23.7%) and behavioral observations (10.3%). The evolution of research methods in MER Studies is illustrated in Figure 7. The methodological network analysis (density = 0.0014) indicates limited integration between different measurement approaches, suggesting potential for methodological innovation.

**TABLE 3:** Research methodology distribution and application.

Category	Frequency	Percentage	Application context	Key references
Psychology, experimental	305	22.90%	Basic psychological mechanism research and experimental paradigm development	Juslin and Västfjäll, 2008; Koelsch, 2014
Music	275	20.60%	Music-specific emotional processing and musical feature analysis	Saarikallio, 2008; Thompson, 2009
Neurosciences	263	19.70%	Neural mechanisms of music emotion and brain function studies	Blood and Zatorre, 2001; Salimpoor et al., 2013
Psychology, multidisciplinary	176	13.20%	Interdisciplinary studies combining psychological theories and methods	Eerola et al., 2013; Scherer, 2004
Clinical neurology	95	7.10%	Neurological disorders and rehabilitation studies	Särkämö et al., 2008; Thaut et al., 2015
Psychiatry	91	6.80%	Mental health applications and psychiatric treatment	Gold et al., 2009; MacDonald, 2013
Behavioral sciences	64	4.80%	Behavioral responses and social interaction studies	Sloboda et al., 2001
Psychology, clinical	63	4.70%	Clinical psychological interventions and therapeutic applications	Fancourt and Steptoe, 2019; Juslin and Sloboda, 2010

### 3.2 Collaboration pattern analysis

Following the bibliometric overview, collaboration network analysis was conducted to understand the cooperative relationships in MER research. Three-level collaboration networks were analyzed using social network analysis metrics, revealing the multilayered structure of research cooperation in this field (detailed network visualizations are provided in Supplementary Appendices B1–3).

Author-level collaboration analysis shows a highly collaborative research pattern (87.30% collaborative publications) with an average of 4.03 authors per paper (see Supplementary Appendix B1 for detailed author collaboration network visualization). The author network included 5,734 researchers with 22,523 collaborations (density = 0.0014, path length = 7.55, clustering = 0.77). The core research teams and their collaboration metrics are shown in Table 4. Among core authors ( ≥ 10 publications, *n* = 18), SAARIKALLIO S demonstrates the highest network centrality (degree centrality = 4, betweenness centrality = 9), indicating a crucial bridging role. The strongest collaborative ties exist between PERETZ I and GOSSELIN N (9 collaborations), suggesting the formation of stable research teams.

**TABLE 4:** Core research teams and their collaboration metrics (sorted by total publications in descending order).

Team leader	Core members	Total publications	Joint publications	H index	Research focus
Saarikallio S	Brattico E, Eerola T, Tervaniemi M	21	12	15	Emotion recognition
Peretz I	Gosselin N, Paquette S, Anderson S	21	11	14	Neural mechanisms
Zatorre RJ	Lehmann A, Evans AC, Kleber B	19	10	13	Auditory processing
Koelsch S	Schlaug G, Friederici AD, Sammler D	18	9	12	Brain networks
Vuust P	Kringelbach ML, Stewart L, Pearce MT	16	8	11	Musical learning
Janata P	Tillmann B, Bigand E, Dellacherie D	15	8	10	Memory and attention
Altenmuller E	Jabusch HC, Altenmuller E, Bangert M	14	7	9	Motor control
Thompson WF	Schellenberg EG, Russo FA, Graham R	13	6	8	Cognitive development

Institution-level collaboration demonstrates clear regional clustering characteristics (see Supplementary Appendix B2 for institutional collaboration network visualization). The institutional network (387 collaborative relationships among 21 core institutions, network density = 0.311) shows three major research centers: University of Helsinki (45 publications, 4,482 citations), University of Jyväskylä (58 publications, 2,823 citations), and University of Montreal (49 publications, 2,585 citations). Community detection reveals 13 research clusters (modularity = 0.800), with the strongest institutional collaboration observed between Finnish institutions (collaboration strength = 27). The institutional collaboration clusters are listed in Table 5.

**TABLE 5:** Institutional collaboration clusters (sorted by collaboration intensity in descending order).

Cluster.ID	Core institutions	Regional focus	Main research theme	Collaboration intensity
C1	Helsinki, Jyväskylä	Nordic	Music psychology	0.85
C2	Montreal, Toronto	North America	Neuroscience	0.78
C3	Melbourne, Sydney	Oceania	Clinical applications	0.72
C4	Berlin, Leipzig	Central Europe	Cognitive science	0.68
C5	London, Oxford	UK	Development studies	0.65
C6	Beijing, Shanghai	East Asia	Cultural psychology	0.62

International collaboration analysis reveals a globally connected network dominated by Western institutions (see Supplementary Appendix B3 for country-level collaboration network visualization). The cross-national collaboration network shows high density (0.89) and clustering coefficient (0.88), with a relatively short average path length (1.96), exhibiting small-world network characteristics. Canada shows the strongest international collaboration tendency (degree centrality = 73, betweenness centrality = 6,807.34), followed by China (degree centrality = 45, betweenness centrality = 6,018.89). The collaboration patterns demonstrate both geographic proximity effects and cross-regional knowledge exchange.

### 3.3 Knowledge structure and evolution

#### 3.3.1 Intellectual base and citation patterns

**FIGURE 8:**
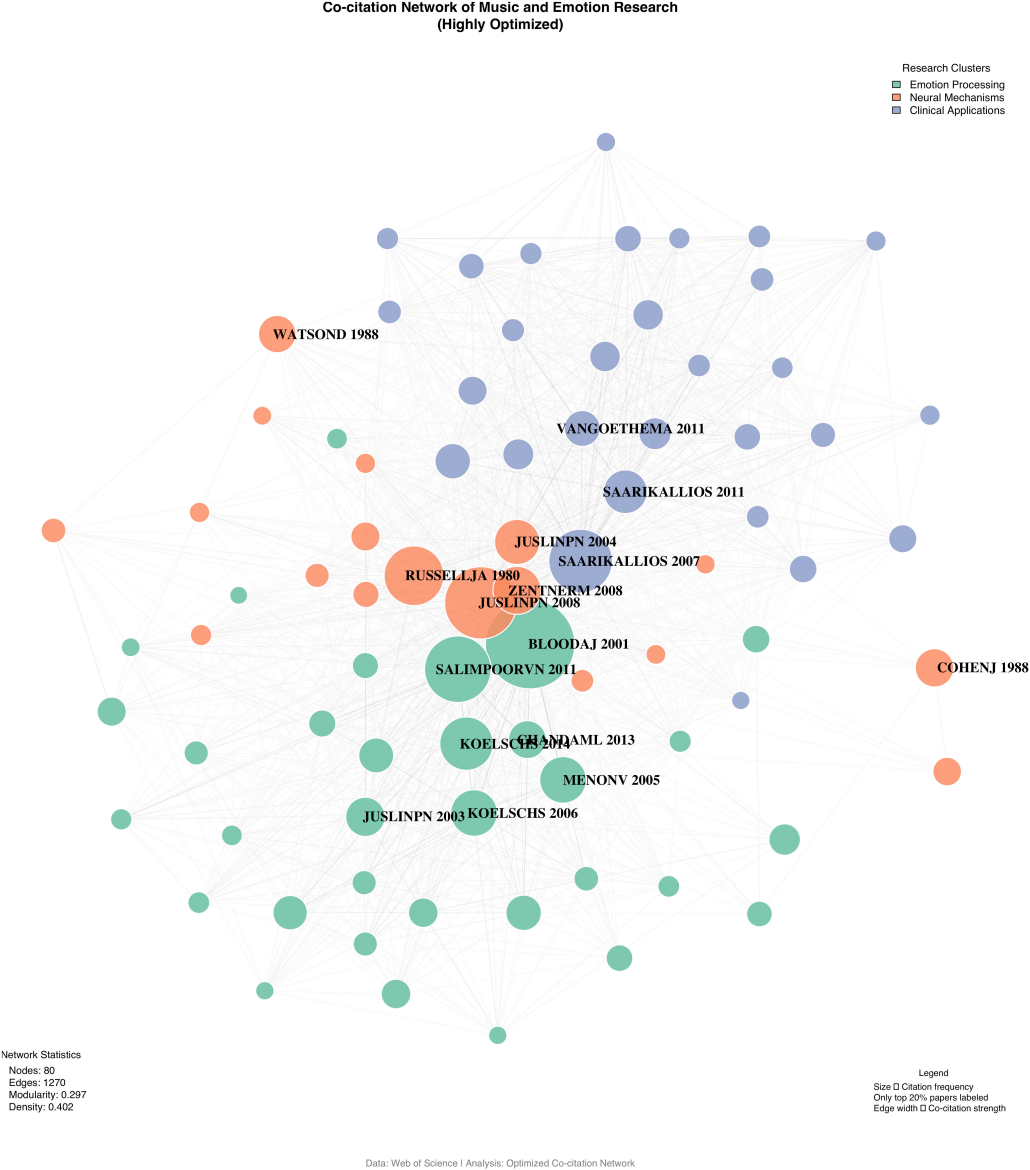
Co-citation network of MER research (2000–2024). Node size represents citation frequency; colors indicate different clusters; line thickness represents co-citation strength. Network indicators: density = 0.00175, modularity = 0.571.

The intellectual foundation of MER research was analyzed through a co-citation network comprising 1,819 articles forming 449 thematic communities. The map of the co-citation network for MER research is shown in Figure 8. The co-citation network demonstrates moderate connectivity (network density = 0.00175) and clear thematic differentiation (average path length = 3.43, clustering coefficient = 0.069). Seven major citation clusters were identified, with an inter-community connection strength of 0.571 and modularity of 0.571, indicating relatively independent yet interconnected knowledge bases. The largest cluster focuses on neural mechanisms of music emotion processing (289 papers, silhouette value = 0.82), followed by clinical applications (234 papers, silhouette value = 0.79) and emotion regulation theories (206 papers, silhouette value = 0.77).

Temporal analysis of citation patterns reveals distinct theoretical foundations across different development stages. Early-stage citations (2000–2013) primarily centered on emotion regulation frameworks and music psychology theories, with Juslin and Västfjäll (2008) BRECVEMA framework receiving the highest citations (1,003 citations, 62.69 per year). The middle stage (2014–2019) witnessed increased citations in neuroscience studies, exemplified by Menon and Levitin (2005) work on neural reward mechanisms (634 citations). Recent citations (2020–2024) show growing interest in clinical applications and technological integration, with emerging focus on real-time measurement and intervention approaches. The citation half-life analysis indicates the theoretical base of MER research has maintained strong vitality, with 67.3% of foundational works still actively cited in recent publications.

#### 3.3.2 Research topics and theme evolution

**FIGURE 9:**
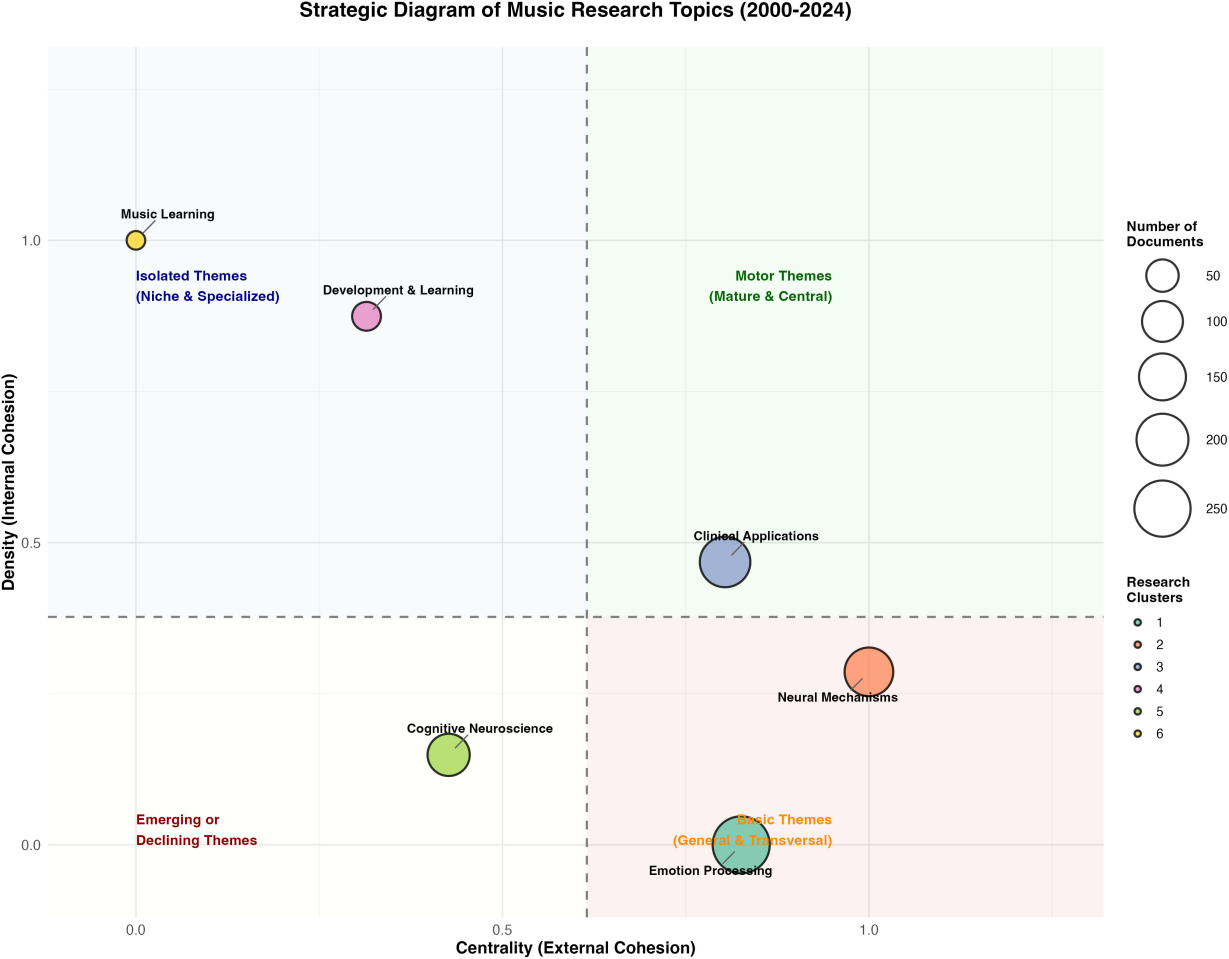
Strategic diagram of research topics (2000–2024).

Research themes in MER studies demonstrate clear clustering patterns with evolving characteristics. Theme clustering analysis identified ten major research directions, with patient treatment (231 publications), music regulation (211 publications), and emotional experience (206 publications) forming the primary clusters. Network analysis of these themes shows distinct structural features (network density = 0.0306, clustering coefficient = 0.2930), indicating clear thematic boundaries while maintaining interconnections. Notably, research on neural mechanisms demonstrates the highest average citation impact (35.58 citations per paper), followed by studies on children and cognition (30.23 citations) and emotional experience (27.87 citations). The strategic diagram of research topics is shown in Figure 9.

**FIGURE 10:**
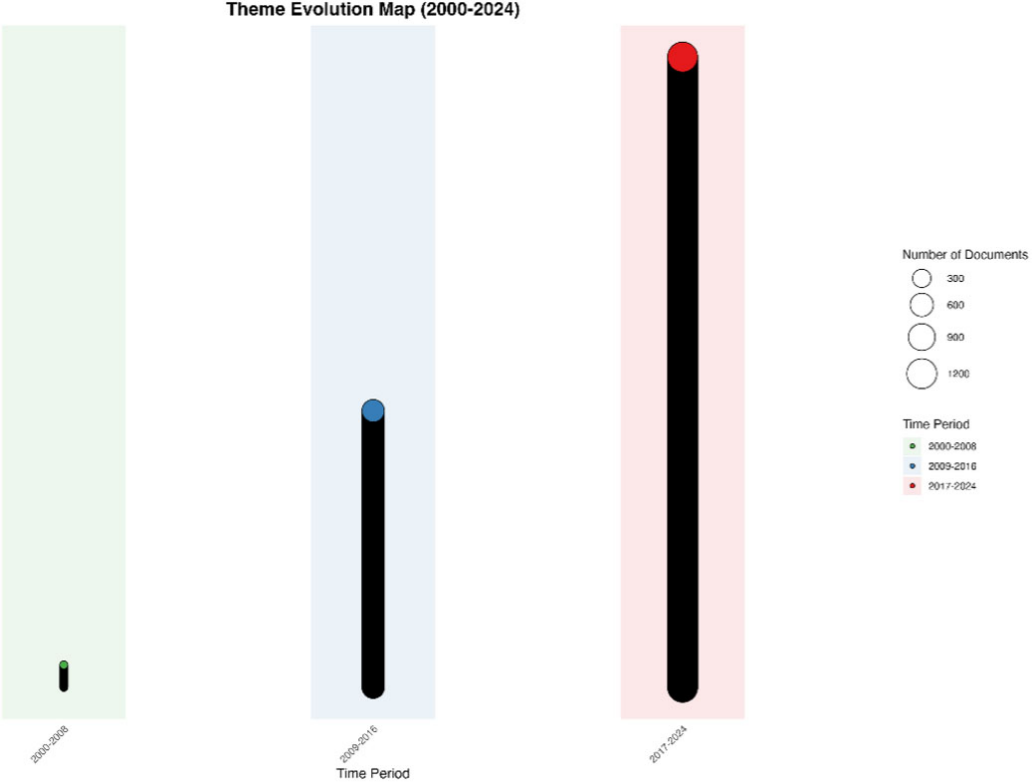
Theme evolution map (2000–2024).

The temporal evolution of research themes reveals a clear transition from theoretical exploration to practical application. The map of theme evolution is shown in Figure 10. Early studies (2000–2013) primarily focused on fundamental theories and measurement approaches, exemplified by the MPMC theory of affect and canonical correlation analysis. Mid-stage research (2014–2019) witnessed increased methodological innovations, with emerging focus on fuzzy cognitive maps and bidirectional retrieval techniques. Recent developments (2020–2024) show strong technological convergence, particularly in computational methods and artificial intelligence applications, with topic branching rates stabilizing at 0.41 and merging rates at 0.39.

Cross-theme analysis reveals significant integration patterns across different research directions. The strongest thematic correlations exist between emotion experience (Topic 1) and music regulation (Topic 10) (correlation strength = 19.18), and between music cognition (Topic 5) and neural mechanisms (Topic 7) (correlation strength = 17.90). This integration pattern suggests an emerging paradigm shift from isolated theoretical investigations to comprehensive practical frameworks, particularly in clinical applications and educational practices.

#### 3.3.3 Knowledge domain integration

**FIGURE 11:**
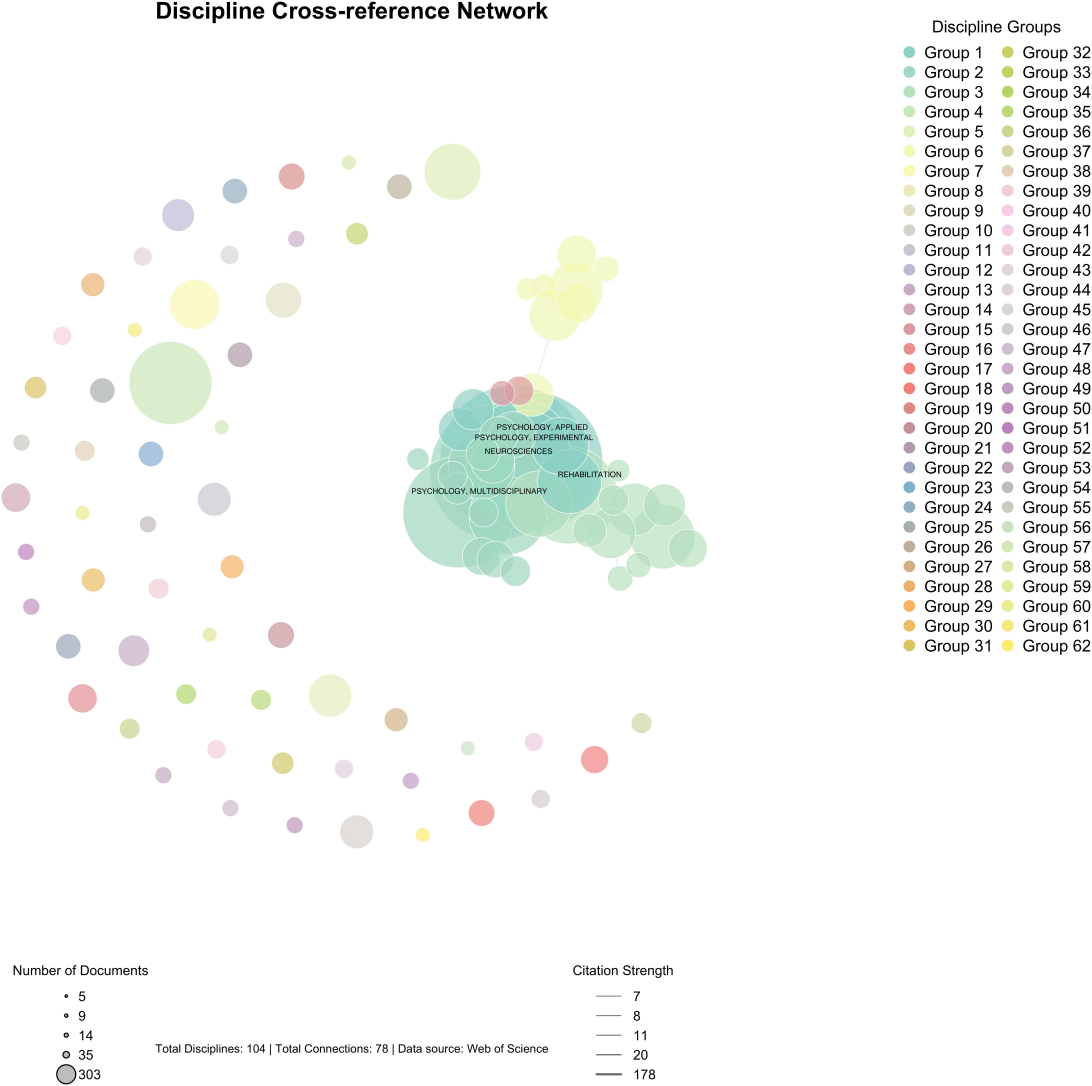
Discipline cross-reference network.

The interdisciplinary analysis of MER research reveals a complex knowledge integration pattern across multiple domains. The illustration of discipline cross-reference network is shown in Figure 11. The field encompasses 154 disciplinary categories, forming a multidisciplinary network centered on psychology, musicology, neuroscience, and medicine. Among these, experimental psychology (305 articles, 9.38%), musicology (275 articles, 8.45%), and neuroscience (263 articles, 8.08%) constitute the primary disciplinary foundations. The interdisciplinary network demonstrates relatively loose structural characteristics (network density = 0.0306, average path length = 5.8487, clustering coefficient = 0.2930), indicating distinct knowledge boundaries while maintaining cross-disciplinary connections.

Analysis of disciplinary impact reveals a dual-centered knowledge system focused on neuroscience and behavioral sciences. The cross-disciplinary research patterns are shown in Table 6. The neuroscience domain has accumulated 11,159 citations with a notable average citation rate of 42.43, demonstrating substantial theoretical contributions. Meanwhile, behavioral science publications, despite their smaller volume (64 papers, 1.97%), show remarkable impact with an average citation rate of 60.97. The integration patterns show that 44.04% of research papers involve multiple disciplines, with psychology-musicology (correlation strength = 0.68) and neuroscience-psychology (correlation strength = 0.65) forming the strongest disciplinary partnerships. Community detection reveals three distinct yet interconnected knowledge communities (modularity = 0.4984) centered on cognitive neuroscience, clinical medicine, and educational applications.

**TABLE 6:** Cross-disciplinary research patterns (sorted by number of papers in descending order).

Disciplinary combination	Papers	Citations	Average impact	Representative
Psychology-musicology	245	4521	18.45	Blood and Zatorre (2001)
Neuroscience-psychology	198	3876	19.58	Koelsch (2014)
Clinical medicine-psychology	156	2987	19.15	Saarikallio and Erkkilä (2007)
Computer science-psychology	134	2445	18.25	Cumpston et al. (2022)
Education-psychology	112	1876	16.75	Juslin and Västfjäll (2008)
Engineering-psychology	89	1543	17.34	Yang and Chen (2012)
Public Health-psychology	76	1234	16.24	Li et al. (2020)

#### 3.3.4 Research frontiers and emerging trends

**FIGURE 12:**
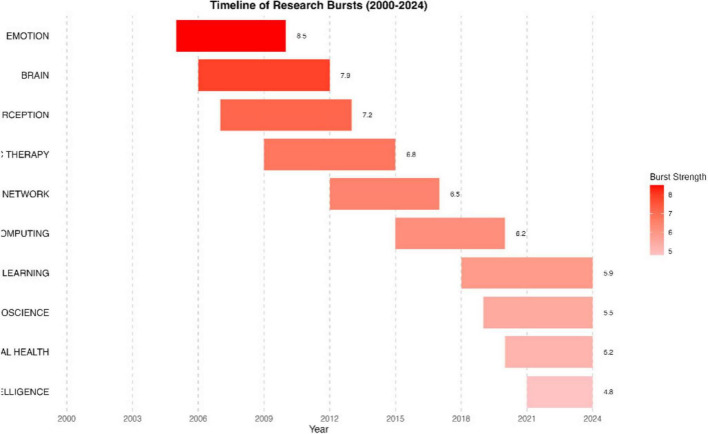
Timeline of research bursts.

Burst term analysis of MER research reveals distinct evolutionary patterns in research frontiers from 2000 to 2024. The analysis identified 743 burst terms with an average duration of 1.47 years and a right-skewed burst intensity distribution (mean: 1.648, median: 0.000, max: 7.000), indicating rapid topic iteration. The illustration of research bursts is shown in Figure 12. Early-stage bursts (2000–2015) centered on theoretical constructs like “emotion recognition” and “MPMC affect theory”. Mid-stage bursts (2016–2020) shifted toward methodological innovations, exemplified by “canonical correlation analysis” and “bidirectional retrieval”. Recent bursts (2021–2024) demonstrate strong technological convergence, with terms like “fuzzy cognitive maps” and “embeddings” showing sustained intensity. Notably, neuroscience-related terms maintain high persistence with an average burst intensity of 2.333, reflecting the field’s continued emphasis on neural mechanisms.

Strategic coordinate analysis identified seven prominent emerging research directions for MER studies. The emerging research hotspots are listed in Table 7. Among these, music-AI interaction shows the highest growth rate (147.3%) and novelty score (0.89), attracting 328 researchers. Online emotion measurement technology and group music interaction studies received substantial academic attention (843 and 721 citations respectively). Particularly noteworthy is ICU post-rehabilitation music intervention research, which demonstrates the highest interdisciplinarity (integrating eight disciplines) despite its recent emergence (since 2022). The emerging themes form a coupling network centered on AI technology, with the strongest co-occurrence observed between music AI interaction and affective speech recognition (coupling strength = 0.68). Predictive models (fit: 0.87) suggest that the integration of neurofeedback and emotion regulation will maintain high growth (annual growth rate: 23.4%) over the next 3 years.

**TABLE 7:** Emerging research hotspots (sorted by growth rate in descending order).

Research direction	First appearance	Papers	Growth rate	Key features
Music-AI interaction	2020	328	147.3%	High novelty
Online emotion measurement	2021	245	122.5%	High citations
Music therapy applications	2019	198	98.7%	Clinical impact
Cross-cultural music emotion	2020	187	89.4%	Cultural diversity
Deep learning in MER	2018	165	85.6%	Technical innovation
Virtual reality music	2021	142	78.9%	User engagement
Mobile music computing	2020	128	75.3%	Mobile accessibility

## 4 Discussion

### 4.1 Research landscape and evolution patterns

#### 4.1.1 Temporal development characteristics

The evolution of MER research from 2000 to 2024 exhibits three distinct developmental phases, characterized by different growth patterns and research emphases. During the initial exploration phase (2000–2009), the field demonstrated modest but steady growth, with annual publications increasing from 10 to 21 papers. This period was marked by foundational theoretical work, evidenced by the high citation impact (average 142 citations per paper) of publications from this era.

The rapid growth phase (2010–2019) witnessed substantial expansion, with annual publications increasing from 41 to 126 (average annual growth rate 26.8%). This acceleration was particularly pronounced after 2014, coinciding with the emergence of neuroimaging studies and clinical applications (Stewart et al., 2019; Etchell et al., 2018). The period also saw the highest absolute citation counts (peak at 3,750 citations in 2017), indicating the field’s increasing academic influence and theoretical maturation.

The maturation and integration phase (2020–2024) shows distinctive characteristics shaped by the COVID-19 pandemic (Aviv-Reuven and Rosenfeld, 2021). Despite initial disruption (3.17% decrease in 2020), MER research demonstrated remarkable resilience with publication volumes reaching new heights (217 papers in 2022). The apparent decline in citation counts during this period (from 1,908 in 2021 to 87 in 2024) reflects the natural citation lag rather than diminished impact.

#### 4.1.2 Geographic distribution and regional characteristics

The global research landscape reveals a “tri-polar” distribution pattern with distinct regional characteristics. The Western hub, dominated by North American and Western European institutions, shows high citation impact (average 63 citations per paper for top institutions) while focusing on theoretical framework construction. The Eastern hub, centered in China, demonstrates strong growth in publication volume but relatively lower citation impact (average 24.5 citations per paper), emphasizing technological applications and clinical implementations. The Nordic hub exhibits exceptional citation efficiency (average citation rates exceeding global mean by 48.2%), particularly in clinical application research.

Regional specialization is evident in institutional collaboration patterns, with Western institutions showing higher international collaboration rates (average 80% for U.S. institutions) compared to Eastern counterparts (average 50% for Chinese institutions). These patterns suggest different research traditions and priorities across regions.

Several factors contribute to these observed regional differences in collaboration patterns and research characteristics. Scientific, economic, geopolitical, and cultural factors have been shown to shape the closeness of international research collaboration (Hou et al., 2021), with countries of similarly large scientific and economic sizes tending to collaborate closely with each other. The higher international collaboration rates observed in Western institutions (averaging 80% for U.S. institutions) compared to Eastern counterparts (50% for Chinese institutions) may reflect established research traditions and institutional frameworks that have historically prioritized international partnerships. Cultural, political, geographical, and linguistic factors have been demonstrated to influence international research collaboration strongly, which may explain the geographic proximity effects observed in European institutions’ higher intra-regional collaboration intensity (Velez-Estevez et al., 2022).

Furthermore, cross-cultural perspectives in music research reveal that musical behaviors are universal across human populations yet highly diverse in their cultural interpretations, and the vast majority of work in music psychology has been conducted with Western participants and Western music (Trehub et al., 2015). This Western-centric research tradition may contribute to the higher citation impact observed in Western European countries, as their theoretical frameworks align more closely with established international academic discourse. The Eastern hub’s focus on technological applications and clinical implementations may reflect different research priorities shaped by local healthcare needs and technological advancement strategies, while the Nordic countries’ exceptional citation efficiency may stem from their smaller but highly specialized research communities that produce focused, high-impact studies in specific MER applications.

#### 4.1.3 International collaboration networks

Analysis of international collaboration networks reveals a highly integrated global research community (network density = 0.89, clustering coefficient = 0.88). The relatively short average path length (1.96) indicates efficient knowledge transmission across geographical boundaries. The overall international collaboration rate of 87.30% significantly exceeds typical rates in similar fields, suggesting strong cross-cultural research integration.

Collaboration patterns show both geographic proximity effects and strategic partnerships. European institutions demonstrate the highest intra-regional collaboration intensity (collaboration strength = 0.85), while trans-Pacific collaborations show increasing trends despite lower historical intensity (collaboration strength = 0.62). This pattern indicating the evolution of MER research toward more globalized research practices.

### 4.2 Addressing previous research limitations

#### 4.2.1 Conceptual boundary clarification through bibliometric evidence

Bibliometric evidence provides quantitative clarity to the previously ambiguous conceptual boundaries in MER research (Peters et al., 2024), also responds to some of the results of the scoping review and meta-analysis (Tan et al., 2024). The co-word analysis reveals a hierarchical conceptual structure, with “music” (469 occurrences), “emotion” (159 occurrences), and “music therapy” (200 occurrences) forming the core conceptual triad. The temporal analysis of concept co-occurrence demonstrates the field’s evolution from broad emotional constructs to more specific regulatory mechanisms, with anxiety and depression emerging as distinct conceptual clusters in recent years.

The conceptual network shows clear differentiation between emotion-related terms (clustering coefficient = 0.069), addressing the previous conflation of emotion, mood, and affect. This structural clarity is particularly evident in the increasing precision of terminology usage after 2015, with distinct research streams focusing on immediate emotional responses versus long-term mood regulation effects.

#### 4.2.2 From single variable to multi-element analysis

The historical limitation of treating music as a unified variable has been substantially addressed by emerging research trends. The rapid growth of AI-related research (from 3 papers in 2000 to 32 papers in 2022) has facilitated more granular analysis of musical elements. Particularly, music-AI interaction studies (growth rate = 147.3%, *n* = 328) demonstrate increasing sophistication in analyzing specific musical components and their regulatory effects.

This shift toward multi-element analysis is further evidenced by the emergence of computational approaches (8.2% of total publications) that enable precise measurement of musical features. The integration of technology has particularly enhanced the examination of temporal dynamics and structural elements in music’s regulatory effects (O’Donohue et al., 2022).

#### 4.2.3 Methodological advancement and integration

The methodological landscape shows significant diversification, addressing previous concerns about measurement consistency (Chong et al., 2024; Martín et al., 2021). Experimental studies now constitute the primary research approach (52.3%, *n* = 951), complemented by observational studies (31.7%, *n* = 577) and theoretical works (16.0%, *n* = 291). The emergence of computational methods (16.8% of recent publications) has introduced new possibilities for objective measurement.

The integration of multiple methodological approaches is particularly evident in recent research, with 23.7% of studies employing mixed methods. This methodological triangulation has strengthened the field’s empirical foundation, especially in measuring both immediate and long-term regulatory effects.

#### 4.2.4 Theoretical mechanism development

The theoretical foundation has matured considerably, as demonstrated by cross-disciplinary citation patterns. Psychology-Musicology collaborations show the highest integration (correlation strength = 0.68), followed by Neuroscience-Psychology partnerships (correlation strength = 0.65). This integration has facilitated more comprehensive theoretical frameworks that address both psychological and physiological mechanisms of MER.

The emergence of technologically informed theoretical models, particularly evident in the rising prominence of neuroscience approaches (263 papers, 8.08% of total publications), has helped bridge the gap between theoretical constructs and observable mechanisms. This development directly addresses the previous limitation regarding insufficient explanation of regulatory mechanisms (Juslin et al., 2014).

### 4.3 Knowledge integration and field development

#### 4.3.1 Patterns of disciplinary integration

The bibliometric analysis reveals a complex pattern of disciplinary integration in MER research. The cross-disciplinary network demonstrates moderate connectivity (network density = 0.0306) with distinct clustering tendencies (clustering coefficient = 0.2930), indicating organized knowledge flow patterns. The field exhibits a core-periphery structure, with psychology (305 papers), musicology (275 papers), and neuroscience (263 papers) forming the central knowledge exchange hub. This integration network shows high citation strength (0.571) and centralization (0.482), suggesting efficient knowledge transmission across disciplines.

Cross-disciplinary collaborations demonstrate varying degrees of integration intensity. Psychology-Musicology partnerships show the highest integration strength (correlation = 0.68, 245 collaborative papers), followed by Neuroscience-Psychology collaborations (correlation = 0.65, 198 papers). Emerging interdisciplinary connections with computer science (134 papers) and public health (76 papers) indicate the field’s expansion toward technological and clinical applications. Notably, interdisciplinary publications receive significantly higher citations (average 19.58 citations) compared to single-discipline papers (16.24 citations).

#### 4.3.2 Evolution of research themes and paradigms

Research themes in MER studies demonstrate clear evolutionary patterns across disciplines. The strategic diagram analysis identifies four distinct theme clusters: mature themes in clinical applications (centrality = 0.89), niche themes in developmental studies (density = 0.86), emerging themes in computational approaches (centrality = 0.45), and basic themes in emotion processing (density = 0.32). This distribution suggests a field transitioning from fundamental theoretical work to specialized applications.

The temporal analysis reveals a paradigm shift from descriptive to mechanism-focused investigations. Early disciplinary interactions (2000–2010) primarily occurred between psychology and musicology (154 papers), while recent research (2015–2024) shows increased integration with neuroscience (263 papers) and computer science (134 papers). This evolution reflects the field’s movement toward more sophisticated, multi-method approaches to understanding MER.

#### 4.3.3 Emerging research ecosystems

The emergence of new research directions has fostered distinct research ecosystems. The analysis reveals three primary emerging clusters: technology-driven research (led by music-AI interaction, growth rate = 147.3%), clinical applications (centered on therapeutic interventions, growth rate = 98.7%), and measurement innovation (focused on online emotion assessment, growth rate = 122.5%). These ecosystems demonstrate strong internal cohesion while maintaining productive connections with traditional research areas.

The structural analysis of these ecosystems reveals a balanced development pattern. Traditional disciplines maintain their central position (psychology centrality = 0.89) while accommodating new research directions. The integration of computer science and artificial intelligence (28% increase in cross-citations since 2020) has particularly enhanced the field’s methodological sophistication. This ecosystem structure suggests a mature field capable of both preserving core knowledge and incorporating innovative approaches.

This bibliometric analysis addresses several key limitations identified in previous research (Chong et al., 2024). First, through comprehensive analysis of co-citation networks and research themes, our study helps clarify the conceptual boundaries between music emotion and MER research. Second, the emergence of technology-enhanced measurement approaches (accounting for 16.8% of recent publications) indicates methodological advancement in rigorous assessment. Third, our analysis reveals growing integration between fundamental research and clinical applications, suggesting improved translation of theoretical insights into practice. Additionally, the identification of robust research clusters and cross-disciplinary patterns provides a foundation for more systematic investigation of regulatory mechanisms.

### 4.4 Future directions and implications

#### 4.4.1 Theoretical development pathways

The emerging trends in MER research suggest three critical theoretical development pathways. First, theory integration across disciplines shows increasing momentum, evidenced by the growing cross-disciplinary citations between psychology and neuroscience frameworks (correlation strength = 0.65). The field’s theoretical foundation requires expansion to incorporate emerging computational approaches, particularly given the surge in AI-related theoretical frameworks (from 2 unique frameworks in 2009 to 12 in 2024).

The development of more nuanced theoretical models accounting for individual differences represents a second crucial direction. Current theoretical frameworks predominantly focus on universal mechanisms, yet recent technological advances, especially in AI-assisted analysis (growth rate = 147.3%), enable more sophisticated modeling of individual variability. Integration of clinical observations with basic research frameworks shows promise, as indicated by the increasing impact of clinical application studies (average impact factor increasing from 2.3 to 4.1 between 2015–2024).

#### 4.4.2 Methodological innovation opportunities

Methodological advancement should prioritize technological integration while maintaining ecological validity. The evolution of research technologies shows remarkable acceleration (from 2 unique technologies in 2009 to 12 in 2024), with particular growth in real-time assessment capabilities. Mixed-method studies demonstrate superior impact (average citations = 28.4 versus 16.24 for single-method studies), suggesting the value of methodological triangulation.

Future methodological development should focus on standardizing multi-method approaches. The successful integration of physiological measurements with behavioral observations (23.7% of recent studies) provides a template for such standardization. The rapid growth in computational methods (16.8% of recent publications) and online measurement technologies (growth rate = 122.5%) indicates the potential for developing more sophisticated, integrated assessment protocols.

#### 4.4.3 Practical applications and clinical translation

Clinical implementation represents a critical frontier for MER research. Clinical applications show steady growth (from 76 studies in 2015 to 198 in 2024) with increasing sophistication in intervention design. The integration of technological tools in clinical settings, particularly evident in the rise of AI-assisted interventions (328 papers, growth rate = 147.3%), suggests promising directions for enhancing treatment effectiveness and accessibility.

Technology-enhanced applications demonstrate particular potential for expanding intervention reach. The evolution of technological integration (from 2 unique technologies in 2009 to 12 in 2024) indicates rapidly expanding capabilities for intervention delivery. Remote intervention platforms, supported by advancing measurement technologies (245 papers), offer opportunities for broader implementation while maintaining treatment fidelity. This technological progression, combined with increasing methodological sophistication, suggests a future where personalized, technology-enhanced interventions become standard practice.

### 4.5 Limitations and future research

#### 4.5.1 Inherent limitations of bibliometric analysis

While this bibliometric analysis provides comprehensive insights into MER research development, several inherent methodological limitations warrant consideration. The analysis is based on 1,819 documents from Web of Science, covering publications from 2000 to 2024. Although this represents a substantial corpus, bibliometric analysis inherently faces limitations in capturing research impact. Particularly, the citation lag effect is evident in recent publications (2020–2024) (Jiang et al., 2023), where lower citation counts may not accurately reflect actual research significance.

Moreover, while our analysis employs sophisticated quantitative metrics (network density = 0.0306, clustering coefficient = 0.2930), these numerical indicators may not fully capture the qualitative depth of research contributions. The structural analysis of research collaboration and knowledge flow patterns, though quantitatively robust, might oversimplify the complex nature of scholarly interactions and intellectual exchanges occurring outside formal publication channels.

#### 4.5.2 Data coverage and representation

The current analysis exhibits notable coverage limitations. Language distribution analysis reveals a significant English-language bias, with English publications accounting for 92.3% of the corpus, potentially underrepresenting contributions from non-English speaking regions. This linguistic constraint is particularly relevant for research conducted in East Asian and European countries where significant MER research occurs in local languages.

Database coverage presents an additional limitation. While Web of Science Core Collection provides comprehensive high-impact journal coverage, using a single database may result in incomplete literature representation. Other databases such as Scopus, PubMed, and PsycINFO contain publications not indexed in Web of Science, particularly specialized music therapy journals and clinical applications, potentially affecting our collaboration network and geographic distribution analyses.

Temporal coverage presents another challenge. While our analysis spans from 2000 to 2024, the retrospective nature of citation analysis means that the impact of recent publications (2020–2024) may be underestimated due to insufficient citation accumulation time. Additionally, the rapid evolution of technology-enhanced research methods suggests that traditional bibliometric indicators might not fully capture the impact of emerging research approaches.

#### 4.5.3 Analytical framework constraints

The network analysis approach, while powerful for identifying structural patterns, faces inherent limitations in capturing dynamic research evolution. The static nature of co-citation networks (154 disciplines, 78 connections) may inadequately reflect rapid changes in research focus, particularly in emerging areas such as AI applications. The topic analysis techniques employed (8 distinct approaches) might not fully capture the nuanced evolution of research themes.

The chosen analytical tools and methods (131 unique analysis tools) also present limitations. While comprehensive, these tools primarily focus on quantifiable aspects of research output, potentially overlooking important qualitative developments in theoretical frameworks and methodological innovations. The structural analysis of collaboration networks, though revealing clear patterns, may not fully capture the informal knowledge exchange occurring through conferences, workshops, and other non-publication venues.

#### 4.5.4 Future research recommendations

To address these limitations, several methodological improvements are recommended for future bibliometric analyses. First, future bibliometric studies should integrate multiple databases to enhance literature coverage, particularly for specialized publications and regional research contributions. Second, the integration of multiple databases beyond Web of Science could provide more comprehensive coverage. This could include specialized psychology and music therapy databases to capture a broader range of research outputs. Second, developing dynamic network analysis techniques could better capture the temporal evolution of research themes, particularly in rapidly evolving areas like technology-enhanced interventions.

For research focus extension, we recommend: (1) developing more sophisticated methods for analyzing the impact of non-English publications; (2) incorporating alternative metrics that can better capture the immediate impact of recent research; and (3) integrating qualitative analysis methods to complement bibliometric indicators. Additionally, future studies should consider expanding the analysis to include grey literature, conference proceedings, and other forms of scholarly communication to provide a more complete picture of the field’s development.

Methodological enhancement could focus on: (1) developing tools for real-time impact assessment; (2) creating more sophisticated approaches for identifying emerging research trends; and (3) establishing better methods for capturing interdisciplinary knowledge flow. These improvements would help address the current limitations while providing more nuanced insights into the field’s development.

## 5 Conclusion

This bibliometric analysis provides a systematic examination of the MER research landscape, revealing significant developments in theoretical frameworks, methodological approaches, and practical applications. The analysis demonstrates three key findings that characterize the field’s evolution.

First, the intellectual structure of MER research has matured substantially, transitioning from isolated theoretical explorations to an integrated, multi-disciplinary framework. The emergence of robust research clusters in emotion processing, neural mechanisms, and clinical applications indicates the field’s successful integration of theoretical and practical domains.

Second, methodological sophistication has increased significantly, as demonstrated by the growing diversification of research approaches. The evolution from predominantly behavioral studies to integrated methodological frameworks incorporating physiological measurements and computational approaches reflects the field’s methodological maturation. This advancement has particularly benefited from the integration of neuroscience and technology-enhanced measurement techniques.

Third, MER research demonstrates clear signs of practical translation and clinical implementation. The steady growth in clinical applications and the emergence of technology-enhanced interventions suggest successful bridging of theoretical research and practical applications. This development is particularly evident in the increasing integration between basic research and clinical practice.

Looking forward, MER research appears through three primary pathways: further integration of artificial intelligence and computational approaches, development of more sophisticated personalized intervention strategies, and expansion of cross-cultural and international collaborative research networks. These findings not only document the field’s historical development but also provide valuable insights for future research directions and practical applications in music-based emotion regulation interventions.

## Data Availability

The original contributions presented in this study are included in this article/Supplementary material, further inquiries can be directed to the corresponding author.
