# Supplementary material for: Music-based emotion regulation: a bibliometric systematic review (2000–2024)
Source: Front Psychol. 2025 Oct 14;16:1565614. doi: 10.3389/fpsyg.2025.1565614 (PMC12558949; doi:10.3389/fpsyg.2025.1565614)
Supplement: Supplementary file 1 [file Data_Sheet_1.docx]

# **Appendix A**

Due to the extensive volume of bibliometric data and supplementary materials, detailed appendices including the complete dataset, R code for analysis, and additional visualizations are made available through a cloud storage repository. These materials can be accessed via insert link as below or by contacting the corresponding author. The comprehensive appendices include:

Complete list of included studies

1. R code for bibliometric analysis and visualization
2. Supplementary figures and detailed network metrics
3. Extended data tables and analysis results

The authors will maintain this data repository for at least five years following publication. For any inquiries regarding access to these materials, please contact the corresponding author at [email address open after proof due to the anonymous review requires].

1. List of Included Studies
2. https://hunnueducn-my.sharepoint.com/:x:/g/personal/201930175079_hunnu_edu_cn/EYS2FWogGQNPn_vmQtE7j30Bm-TOpUpzkMKXYYHQ5wcBZw?e=3jHozs
3. Code for Bibliometric Analysis

https://hunnueducn-my.sharepoint.com/:f:/g/personal/201930175079_hunnu_edu_cn/Ev-lYN3kt3lNukyLrWq7aw8Bi08KmzrKkUtblDFu-AHtCg?e=wDLLA5

1. Additional Network Analyses & Supplementary Tables

<https://hunnueducn-my.sharepoint.com/:f:/g/personal/201930175079_hunnu_edu_cn/Eg6TKN2tKExGlzWl9qu_hLAB7d-tvAC1g7rvbaLXdKMjAQ?e=WfTnyA>

# **Appendix Tables**

**Supplementary Table S1: Complete Author Information for Top 10 Highly Cited Papers**

| **Rank** | **Complete Author List** | **Corresponding Author(s)** |
| --- | --- | --- |
| 1 | Juslin PN, Västfjäll D | Juslin PN |
| 2 | Menon V, Levitin DJ | Menon V |
| 3 | Naseer N, Hong KS | Hong KS |
| 4 | Zentner M, Eerola T | Zentner M |
| 5 | Chanda ML, Levitin DJ | Chanda ML |
| 6 | Särkämö T, Tervaniemi M, Laitinen S, Forsblom A, Soinila S, Mikkonen M, Autti T, Silvennoinen HM, Erkkilä J, Laine M, Peretz I, Hietanen M | Särkämö T |
| 7 | Greenlee H, Dupont-Reyes MJ, Balneaves LG, Carlson LG, Cohen LE, Deng G, Johnson JA, Mumber M, Seely D, Zick SM | Carlson LG |
| 8 | Boyce SM, Tripathy D, Koelsch S | Koelsch S |
| 9 | Barrett FS, Grimm KJ, Robins RW, Wildschut T, Sedikides C, Janata P | Robins KJ |
| 10 | Lehrner J, Marwinski G, Lehr S, Johren P, Deecke L | Lehrner J |

**Note:** Author names are listed as they appear in the original publications. Corresponding authors are identified based on the original paper's contact information.

**Supplementary Table S2: Complete Journal Information and Impact Metrics**

| **Rank** | **Complete Journal Name** | **Journal Abbreviation** | **Impact Factor*** | **Publisher** | **Category** |
| --- | --- | --- | --- | --- | --- |
| 1 | Behavioral and Brain Sciences | Behav Brain Sci | 20.6 | Cambridge University Press | Psychology/Neuroscience |
| 2 | NeuroImage | NeuroImage | 5.9 | Elsevier | Neuroimaging |
| 3 | Frontiers in Human Neuroscience | Front Hum Neurosci | 2.9 | Frontiers Media | Neuroscience |
| 4 | Proceedings of the National Academy of Sciences | PNAS | 12.8 | National Academy of Sciences | Multidisciplinary |
| 5 | Trends in Cognitive Sciences | Trends Cogn Sci | 21.2 | Elsevier | Cognitive Science |
| 6 | Brain | Brain | 14.5 | Oxford University Press | Neurology |
| 7 | CA: A Cancer Journal for Clinicians | CA Cancer J Clin | 286.1 | American Cancer Society | Oncology |
| 8 | Trends in Cognitive Sciences | Trends Cogn Sci | 21.2 | Elsevier | Cognitive Science |
| 9 | Emotion | Emotion | 3.4 | American Psychological Association | Psychology |
| 10 | Physiology & Behavior | Physiol Behav | 3.0 | Elsevier | Behavioral Sciences |

# **Appendix B**

**
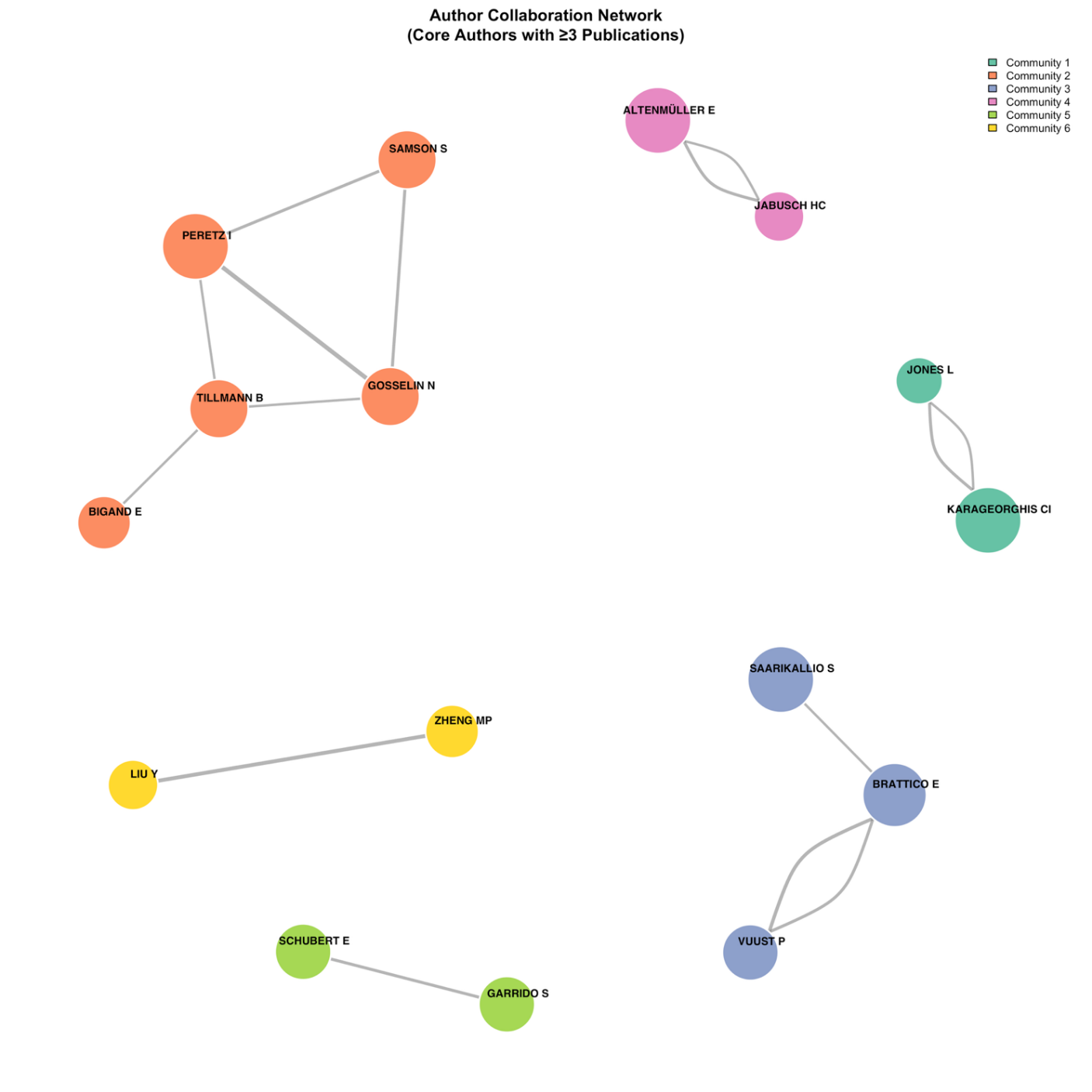
**

**B1. Author Collaboration**

**
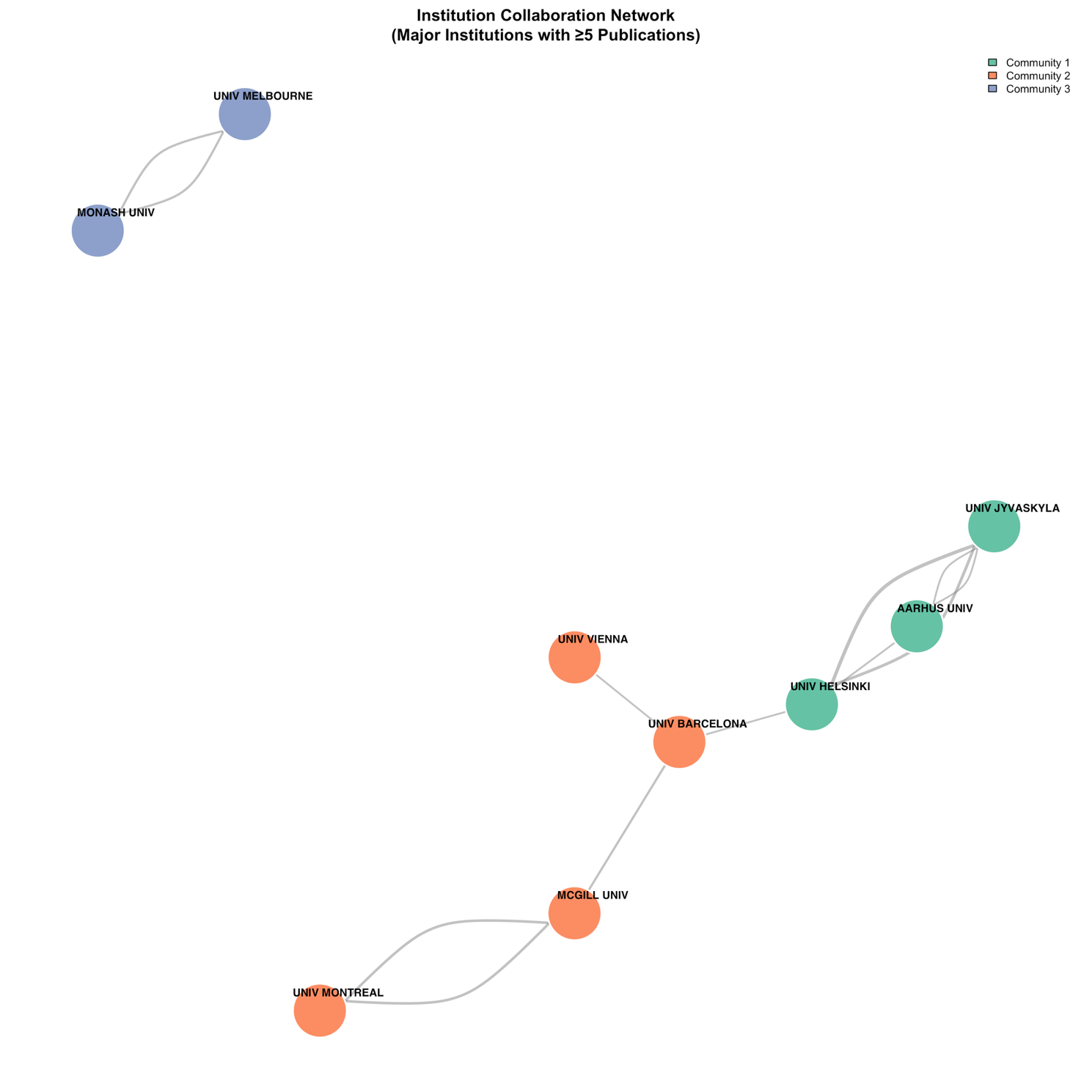
**

**B2. Institutional Collaboration Network**

**
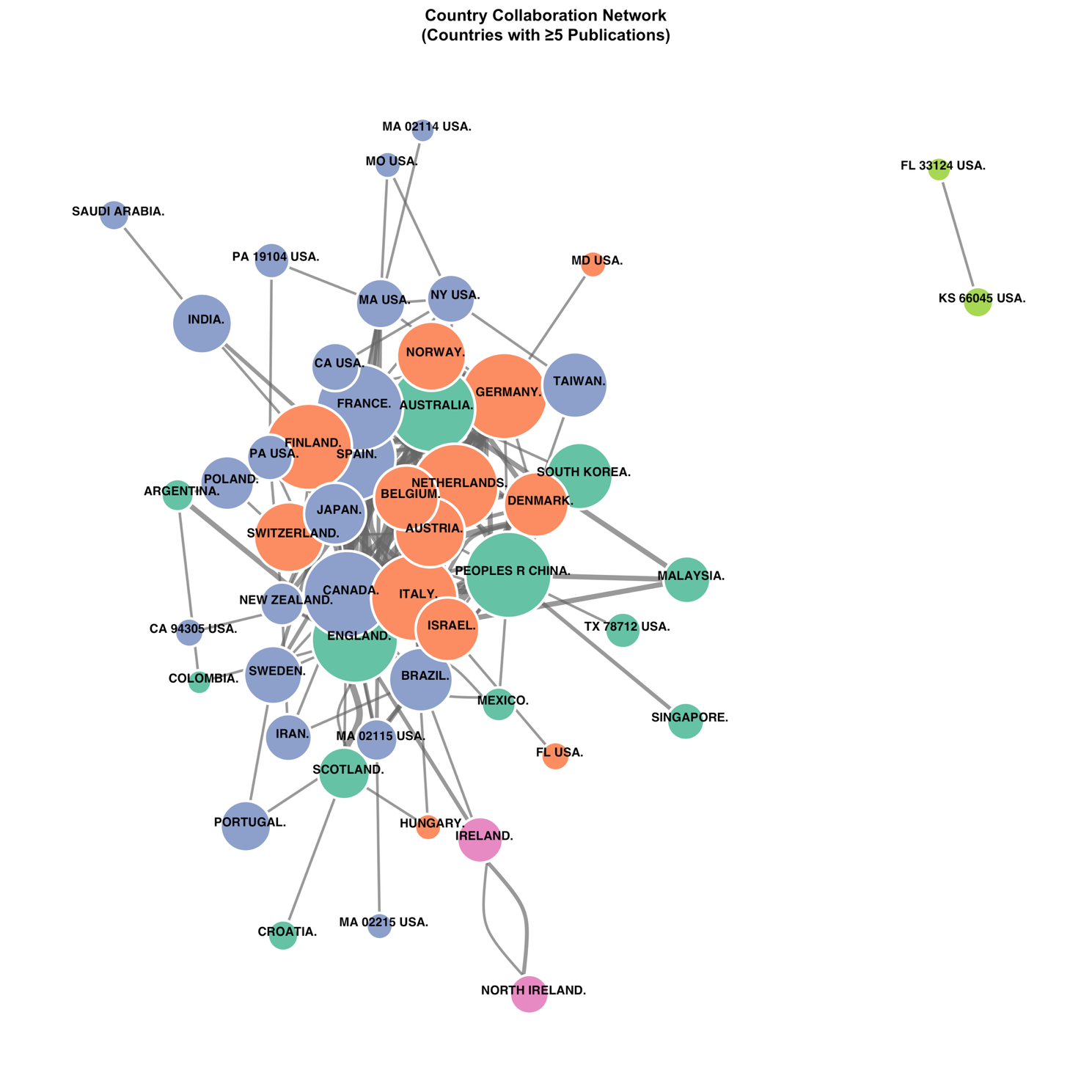
**

**B3. Country-level Collaboration Network**
